# Germ‐free mice are not protected against diet‐induced obesity and metabolic dysfunction

**DOI:** 10.1111/apha.13581

**Published:** 2020-12-06

**Authors:** Chiara H. Moretti, Tomas A. Schiffer, Xuechen Li, Eddie Weitzberg, Mattias Carlström, Jon O. Lundberg

**Affiliations:** ^1^ Department of Physiology and Pharmacology Karolinska Institutet Stockholm Sweden; ^2^ Beijing Key Laboratory of New Drug Mechanisms and Pharmacological Evaluation Study Institute of Materia Medica Chinese Academy of Medical Science & Peking Union Medical College Beijing China; ^3^ Department of Perioperative Medicine and Intensive Care Karolinska University Hospital Stockholm Sweden

**Keywords:** diabetes, diet, germ‐free mice, metabolic syndrome, microbiota, obesity

## Abstract

**Aim:**

Studies in the past 15 years have highlighted the role of the gut microbiota in modulation of host metabolism. The observation that germ‐free (GF) mice are leaner than conventionally raised (CONV) mice and their apparent resistance to diet‐induced obesity (DIO), sparked the interest in dissecting the possible causative role of the gut microbiota in obesity and metabolic diseases. However, discordant results among studies leave such relationship elusive.

In this study, we compared the effects of chronic Western diet (WD) intake on body weight and metabolic function of GF and CONV mice.

**Methods:**

We fed GF and CONV mice a WD for 16 weeks and monitored body weight weekly. At the end of the dietary challenge, the metabolic phenotype of the animals was assessed. Muscle carnitine palmitoyltransferase I (CPT1) and liver AMPK activation were investigated.

**Results:**

Both GF and CONV mice gained weight and developed glucose intolerance when fed a WD. Moreover, WD feeding was associated with increased adipose tissue inflammation, repressed hepatic AMPK activity, fatty liver and elevated hepatic triglycerides in both groups of mice. Enhanced fatty acid oxidation in the GF mouse is one of the proposed mechanisms for their resistance to DIO. The GF mice in this study showed higher CPT1 activity as compared to their CONV counterparts, despite not being protected from obesity.

**Conclusions:**

We provide evidence that the microbiota is not an indispensable factor in the onset of obesity and metabolic dysfunction, suggesting that the relationship between gut bacteria and metabolic diseases needs further exploration.

## 1 INTRODUCTION

Obesity has increased at a fast pace in the past decades, becoming a health issue and social burden of great proportions. Importantly, obesity entails a broad spectrum of metabolic, cardiovascular, renal and musculoskeletal pathologies, leading to severe physical disabilities.^1^ It is also cause of social disability, as it often associates with poor mental health, unemployment and lowered quality of life, ultimately resulting in reduced life expectancy.^2^, ^3^ As of today, prevention and treatment strategies have not proven effective.

A long‐term energy imbalance is recognized as the main contributor to obesity.^4^ In recent years, the gut microbiota has been identified as a regulator of energy metabolism, mediating the response of the host to the diet.^5^, ^6^ This hypothesis originated from the observation that germ‐free (GF) mice, which carry no bacteria whatsoever, are leaner compared to their conventional (CONV) counterparts with a normal microbiota. Moreover, the same lean GF mice colonized with the gut microbiota of CONV mice, gain body fat and show decreased insulin sensitivity.^6^ Later, the same group and others reported lack of an obese phenotype in GF mice fed obesogenic diets^7^, ^8^, ^9^ and described obesity‐specific alterations of the gut microbiome.^10^, ^11^ These studies added the notion that a Western diet (WD) can change the microbial ecology of the gut, likely selecting bacterial communities with increased capacity to harvest the carbohydrates found in the diet and produce short‐chain fatty acids that are readily stored by the host.^10^

This considered, an important question to address is whether this association is causal. Studies using faecal microbial transplantation showed that the obese phenotype can be transferred, via the gut microbiota, from *ob/ob* mice^10^ and obese individuals to GF mice.^12^ Based on these findings, causality has been widely discussed across the scientific literature.^13^, ^14^, ^15^, ^16^, ^17^

However, a few reports have indicated that GF mice are not generally resistant to diet‐induced obesity (DIO).^18^, ^19^, ^20^, ^21^ Moreover, although an increased *Firmicutes* to *Bacteroidetes* ratio is generally associated with increased energy harvest capacity and obesity,^10^, ^22^ other studies could not reproduce such correlations.^23^, ^24^, ^25^, ^26^ Differences in the composition of the high‐fat (HF) diets,^21^ as well as control diets^27^ and mouse strains used in these studies, have been proposed as an explanation for such discordant results. This considerable number of contrasting findings, while highlighting the complexity and multifactorial nature of obesity, also suggests that the gut microbiota might not be the protagonist in the pathogenesis of this disease, but rather one of several players.

In this study, we aimed at clarifying whether the gut microbiota is indeed required for the development of DIO and metabolic syndrome. We fed male mice of the widely used strain C57BL/6J, the same WD used in the original study that first indicated obesity resistance of GF mice.^7^ We then monitored body weight, food and water consumption during 16 weeks of dietary challenge. At the end of this period, we verified the obese phenotype by investigating body composition, glucose tolerance and fasting glucose levels, as well as hepatic triglycerides levels and liver steatosis. At the molecular level, we investigated the consequences of a chronic WD feeding on AMP‐protein kinase (AMPK) activation, which is known to be repressed by a HF diets^28^, ^29^ as well as adipose tissue inflammation, typically elevated in diabetes and obesity.^30^, ^31^

Moreover, increased fatty acid oxidation is one of the suggested mechanisms of DIO resistance in GF mice^7^ and, therefore, we measured CPT1 activity in gastrocnemius muscles of both CONV and GF mice following the dietary challenge.

We show that GF mice progressively gain weight during 16 weeks on a WD. In addition to obesity, GF mice developed impaired glucose tolerance and fasting hyperglycaemia similarly to CONV mice. Despite being susceptible to DIO, the GF mice in this study also showed higher CPT1 activity in gastrocnemius muscle compared to CONV mice.

## 2 RESULTS

### 2.1 Germ‐free mice gain weight when fed a Western diet

**FIGURE 1:**
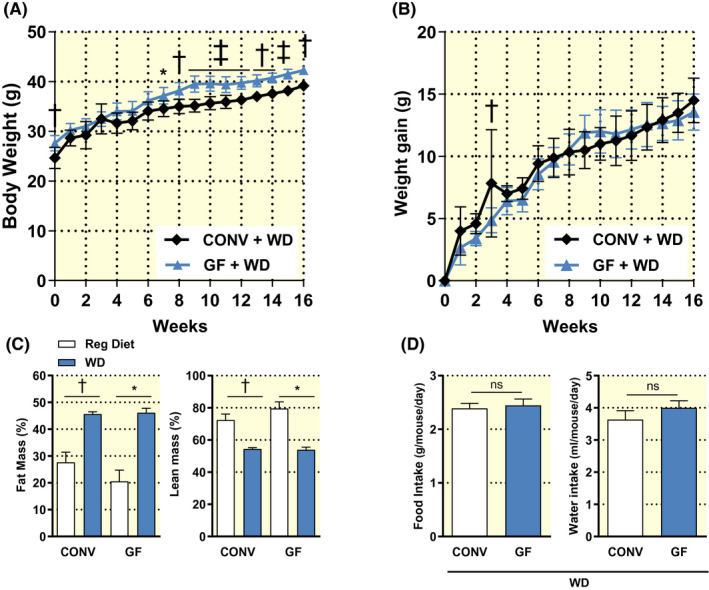
Body weight (A) and body weight gain from baseline (B) of conventional (CONV; n = 6) and germ‐free mice (GF; n = 10) during 16 weeks on a Western diet (WD). Body composition (C) and cumulative food and water intake (D) of mice fed a regular rodent diet (GF n = 3; CONV n = 4) or a WD for 16 weeks (GF n = 6; CONV n = 6). Data are shown as mean ± SD (A; B) and ± SEM (C; D). *p* values were calculated with two‐way ANOVA (A; B), Mann‐Whitney test for comparisons of CONV + Reg Diet vs CONV + WD; GF + Reg Diet vs GF + WD; CONV + WD vs GF + WD (C) or t‐test (D). *P < .05; ^†^ *P* < .01; ^‡^ *P* < .001

The primary aim of this study was to verify whether the absence of host microbiota is protective against DIO. We fed aged‐matched GF and CONV mice a WD for 16 weeks and monitored body weight, food and water intake weekly. After 7 weeks, we noted a significantly higher body weight of GF mice compared to that of CONV controls (Figure 1A). This difference was maintained until the end of the dietary challenge, when GF and CONV mice had gained on average 13.6 ± 1.5 g and 14.7 ± 1.7 g respectively (Figure 1B). Moreover, as compared to GF and CONV mice fed a regular rodent diet, chronic WD consumption resulted in abnormal body mass composition in both GF and CONV groups, with significantly increased fat mass and decreased lean mass (Figure 1C). No differences in cumulative food and water intake were noted (Figure 1D).

### 2.2 Germ‐free and conventional mice develop impaired glucose metabolism when fed a Western diet

Previous studies have shown that GF mice fed a HF diet have lower fasting blood glucose and circulating insulin and they are protected from glucose intolerance.^8^ Therefore, we sought to verify whether the obese phenotype observed in our GF mice was accompanied by perturbation of glucose metabolism.

**FIGURE 2:**
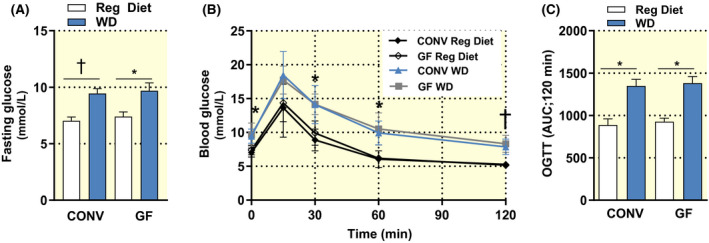
Fasting blood glucose (A); glucose tolerance test curve (B) and area under the curve (C) of conventional (CONV) and germ‐free (GF) mice fed a regular rodent diet (GF n = 3; CONV n = 4) or a Western diet (WD) for 16 weeks (GF n = 6; CONV n = 6). Data are shown as mean ± SEM *P* values were calculated with two‐way ANOVA (A) or Mann‐Whitney test (B; C). In panel B, *Indicates *P* < .05 CONV + Reg Diet vs CONV + WD at 0, 30 and 60 min and GF + Reg Diet vs GF + WD at 60 min; ^†^Indicates *P* < .01 CONV + Reg Diet vs CONV + WD and GF + Reg Diet vs GF + WD at 120 min

Following the dietary challenge, GF and CONV mice had elevated fasting glucose (Figure 2A). These same groups of mice showed equally impaired glucose tolerance (Figure 2B,C) upon oral administration of glucose. This diet‐driven effect is clear from a comparison with CONV and GF mice fed a regular rodent diet.

### 2.3 Germ‐free and conventional mice develop liver steatosis and adipose tissue inflammation on a Western diet

**FIGURE 3:**
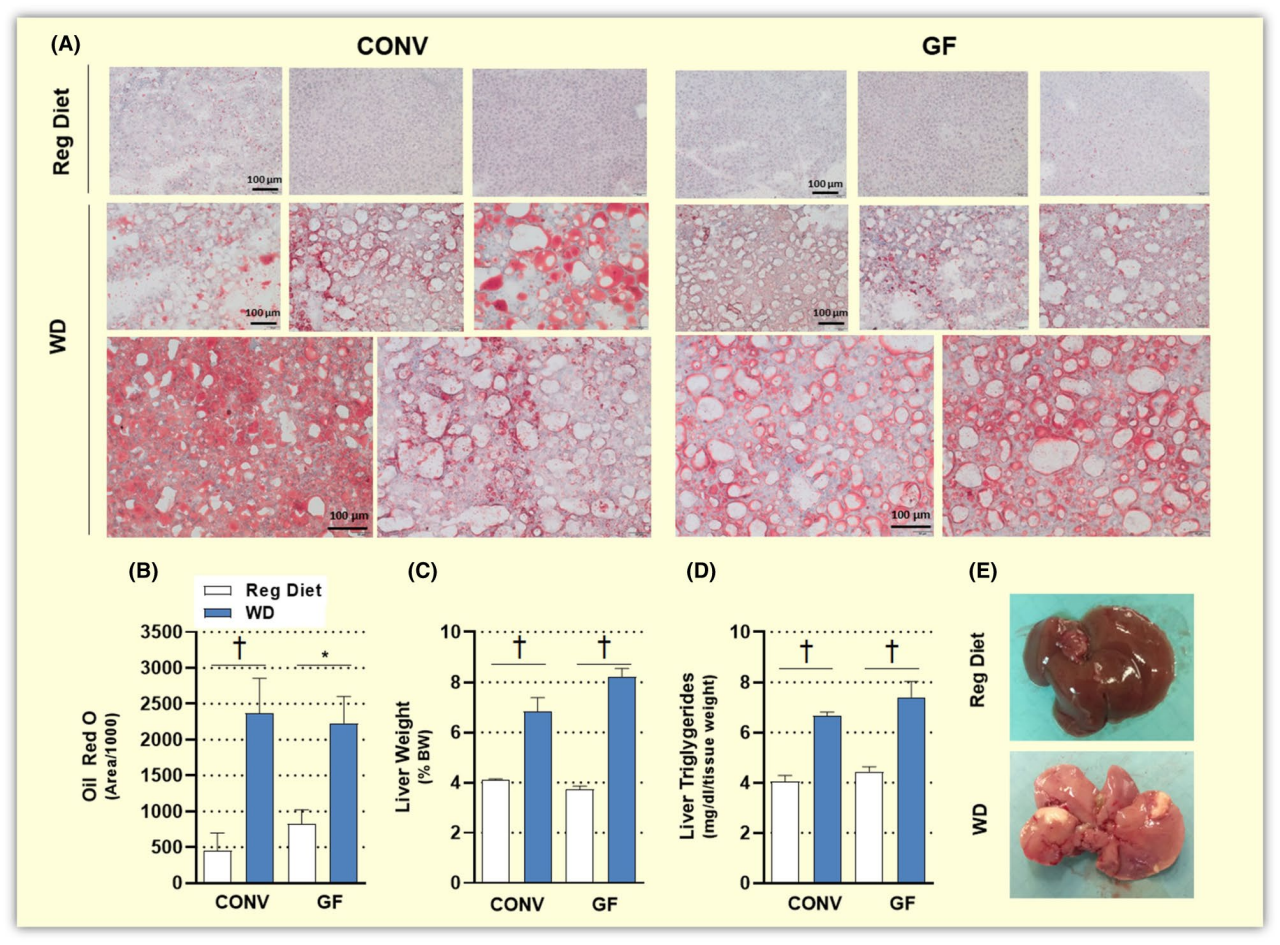
Representative pictures of hepatic tissue stained with Oil Red O (A; magnification 20X, scale bars: 100 µm), quantification of Oil Red O stained area (B), liver weight (C) and liver triglycerides (D) of conventional (CONV) and germ‐free (GF) mice fed a regular diet (GF n = 3‐7; CONV n = 4) or a Western diet (WD) for 16 weeks (GF n = 6; CONV n = 6). Representative pictures of livers from a control mouse on a regular diet (top) and a GF mouse on a Western diet for 16 weeks (bottom; E). Data are shown as mean ± SEM. *p* values were calculated with Mann‐Whitney test for comparisons of CONV + Reg Diet vs CONV + WD; GF + Reg Diet vs GF + WD; CONV + WD vs GF + WD. *P < .05; ^†^ *P* < .01

Mice on a HF diet are known to develop fatty liver already after 2 weeks.^32^ However, GF mice fed the same diet were reported to accumulate less lipids in their liver^8^ and to be protected from liver steatosis.^33^ To verify if this feature of the metabolic syndrome had developed in our GF mice, we measured hepatic triglycerides levels and looked at histological signs of liver steatosis. In both GF and CONV mice fed a WD, livers were significantly heavier than those of GF and CONV controls (Figure 3C). Concomitantly, both groups of mice in this study showed significantly increased hepatic triglycerides, as compared to untreated controls (Figure 3D). This evidence was supported by macroscopic examination of the livers first (Figure 3E) followed by histologic analyses, which revealed steatotic changes in the hepatic tissue upon WD feeding (Figure 3A,B).

**FIGURE 4:**
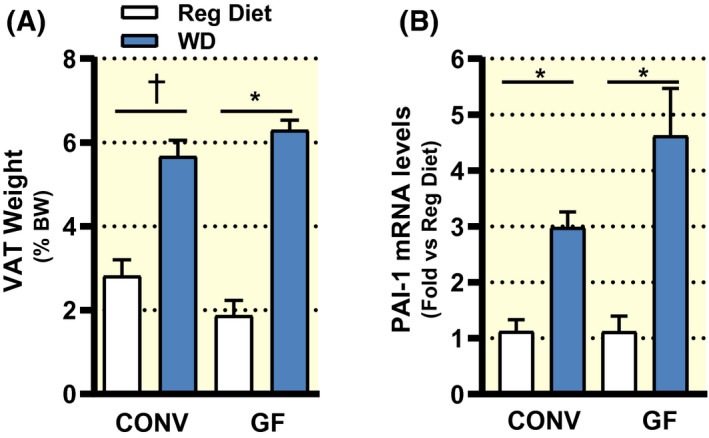
Weight of visceral adipose tissue (A; VAT) of conventional (CONV) and germ‐free (GF) mice fed a regular rodent diet (GF n = 3; CONV n = 4) or a Western diet (WD) for 16 weeks (GF n = 6; CONV n = 6). Relative mRNA levels of the inflammation marker PAI‐1 detected in VAT of CONV and GF mice on a regular diet (CONV n = 5; GF n = 4) or a Western diet (CONV n = 6; GF n = 6) for 16 weeks (B). Data are shown as mean ± SEM. *p* values were calculated with Mann‐Whitney test for comparisons of CONV + Reg Diet vs CONV + WD; GF + Reg Diet vs GF + WD; CONV + WD vs GF + WD. *P < .05; ^†^ *P* < .01

In addition, consumption of a WD significantly increased the weight of visceral adipose tissue (VAT) in both CONV and GF mice (Figure 4A), in agreement with the body composition analyses showed in Figure 1C. Adipose tissue inflammation is crucial in the onset and progression of metabolic disorders and the intestinal microbiota has been indicated as a fuelling factor of metabolic inflammation.^34^ Plasminogen activator inhibitor‐1 (PAI‐1) is an adipokine that is increased in human obesity^35^ and its gene expression levels could be reduced by antibiotic treatments in mice fed a HF diet.^36^ Nonetheless, in this study, we found significantly increased mRNA levels of PAI‐1 in visceral adipose tissue of both CONV and GF mice fed a WD, as compared to their counterparts on a regular diet (Figure 4B).

**FIGURE 5:**
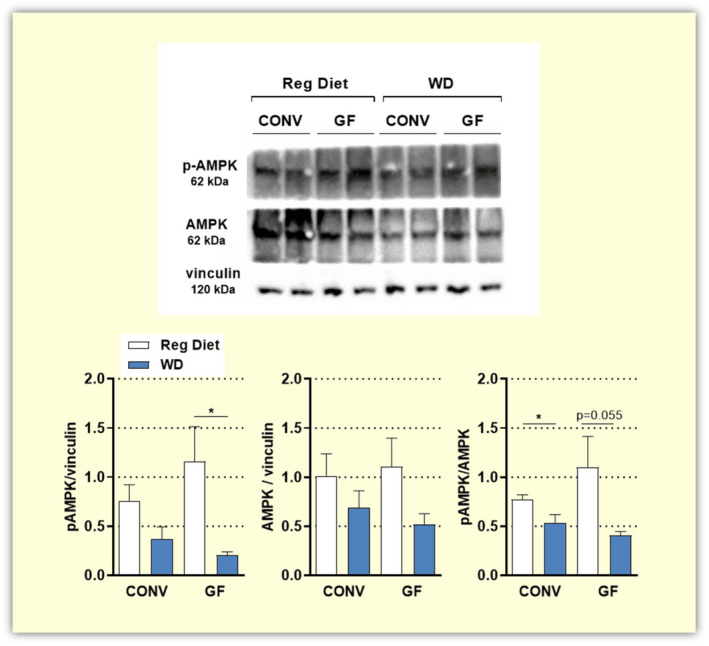
Protein levels of pAMPK and AMPK detected by Western blot in liver lysates of conventional (CONV n = 6) and germ‐free (GF n = 6) mice fed a regular diet or a Western diet (WD) for 16 weeks. Data are shown as mean ± SEM.*p* values are calculated with *t* test for comparisons of CONV + Reg Diet vs CONV + WD; GF + Reg Diet vs GF + WD; CONV + WD vs GF + WD; CONV + Reg Diet vs GF + Reg Diet *P < .05

To further investigate the effects of a WD in GF mice at the molecular level, we studied the activation of hepatic AMP‐activated protein kinase (AMPK), an important sensor of energy status. In fact, reduced AMPK activity has been strongly associated with metabolic diseases including obesity, type 2 diabetes (T2D) and non‐alcoholic fatty liver disease (NAFLD).^37^, ^38^ Previous research indicated increased pAMPK (active) in GF mice fed a WD, as compared to colonized animals. Therefore, the authors suggested that the microbiota would favour the inactive AMPK state, contributing to fat storage and the onset of obesity.^7^ In light of the obese phenotype and metabolic dysfunction developed by the GF mice in the present study, we anticipated that AMPK activity would be suppressed by a protracted WD feeding to the same extent in GF and CONV mice. Following the analysis of pAMPK and AMPK protein levels, we show that consumption of a WD reduced AMPK activation also in the absence of a microbiota. Moreover, no statistically significant differences in AMPK activation were noted between GF and CONV mice when these were fed either a regular diet or a Western diet (Figure 5).

### 2.4 CPT1 activity in skeletal muscle of germ‐free mice is elevated despite the obese phenotype

**FIGURE 6:**
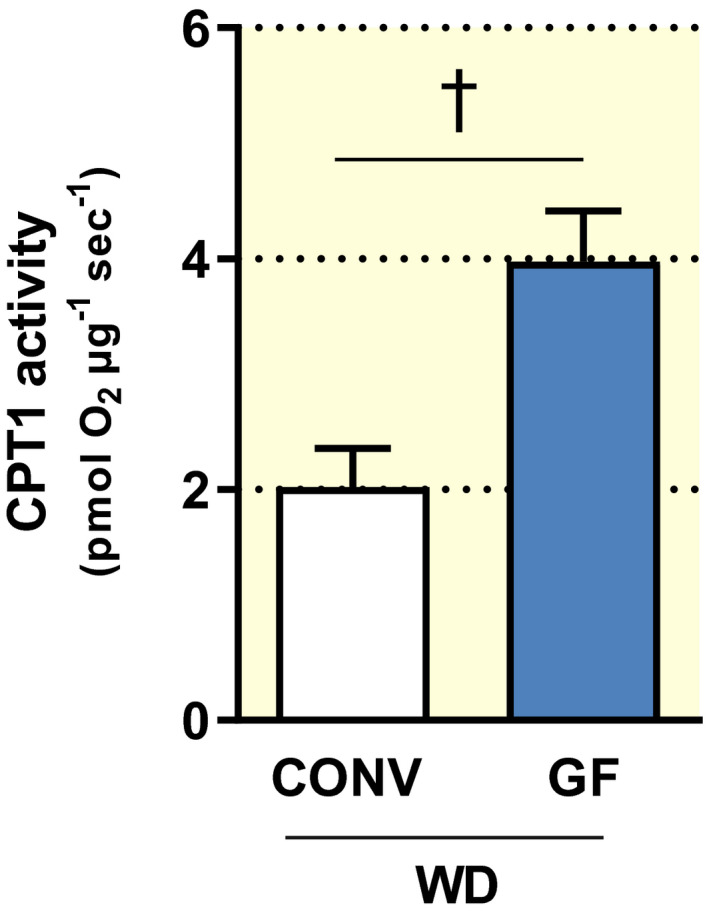
Carnitine palmitoyltransferase I (CPT1) activity in mitochondria isolated from gastrocnemius muscle of conventional (CONV; n = 5) and germ‐free (GF; n = 7) mice after 16 weeks on a Western diet (WD). CPT1 activity was detected by high‐resolution respirometry and normalized to the total protein content in each sample. Data are shown as mean ± SEM *P* value was determined by *t* test. ^†^ *P* < .01

The mitochondrial enzyme CPT1 is responsible for the translocation of free fatty acids from the cytosol to the inner mitochondria. As such, CPT1 represents the rate‐limiting step in long‐chain fatty acid oxidation. From a comparison between GF and colonized mice on a WD, it was proposed that the microbiota might favour greater inhibition of CPT1 in muscle and liver, as a consequence of reduced AMPK activity. This would lead to reduced fatty acid oxidation and ultimately promote fat storage.^7^ In light of our findings presented above, which are in contrast with the same original study,^7^ we decided to investigate fatty acid oxidation in gastrocnemius muscle of GF and CONV mice following 16 weeks of WD feeding. As previously indicated,^7^ we found significantly elevated CPT1 activity in GF compared to CONV mice (*p* = 0.008; Figure 6).

## 3 DISCUSSION

Here, we show that energy harvest from the diet and fat storage are independent of the gut microbiota. In the present study, GF and CONV mice fed a WD developed obesity, glucose intolerance, liver steatosis and adipose tissue inflammation to a similar extent. These results are in contrast to the currently accepted idea that GF mice are resistant to DIO, as first reported in 2007.^7^ These pioneering studies triggered the next 15 years of research investigating a causal role of the gut microbiota in obesity and ultimately in regulating host metabolism. Nevertheless, such direct link remains unproven. In fact, at least four studies have now challenged this hypothesis, showing that the absence of host microbiota does not necessarily correspond to obesity resistance.^18^, ^19^, ^20^, ^21^ Such contrasting findings have been attributed to differences in diet composition ^19^, ^21^ and the mouse strains used.^20^

In this study, we fed GF and CONV C57BL/6J mice the same WD that was used in the first report of DIO resistance in GF mice.^7^ We found that body weight of both GF and CONV mice increased progressively over 16 weeks of dietary challenge, eventually leading to an obese phenotype. This result is supported by an equally increased fat mass in GF and CONV mice fed a WD, as compared to animals on a regular diet.

Obesity is recognized as the greatest risk factor for T2D, with over 80% of diabetic subjects being overweight or obese.^39^ Accumulating evidence implicates the gut microbiota in T2D, with changes in gut microbial profile^40^, ^41^, ^42^ and elevated endotoxemia driven by microbial products, *i.e*. LPS^43^, ^44^, ^45^ as the main proposed mechanisms. In support of this idea, antibiotic‐treated obese mice^46^ as well as HF diet‐fed GF mice have enhanced insulin sensitivity as compared to CONV mice.^8^ Nonetheless, our results indicate impaired glucose tolerance and elevated fasting blood glucose in both GF and CONV mice fed a WD, with no differences noted between the two groups. Supporting our results, in a recent report by Logan et al, GF Swiss Webster mice also show impaired glucose tolerance after 10 weeks of HF diet feeding.^20^

As a consequence of its impact on hepatic glucose and lipid metabolism, the gut microbiota has been implicated in the onset and progression of non‐alcoholic fatty liver disease (NAFLD), a complication of obesity and T2D.^47^ Studies in GF animals have reported lower hepatic lipid accumulation^8^ and resistance to liver steatosis^33^ upon HF feeding. Moreover, colonization of GF animals with gut microbial communities of hyperglycaemic and hyperinsulinemic CONV mice induced NAFLD in the recipients.^48^ In contrast to this, our GF mice showed elevated visceral adipose tissue, elevated hepatic triglycerides as well as fatty liver at the end of the WD challenge. Supporting this evidence, hepatic AMPK activity was repressed by a WD in the GF mice. Reduction in AMPK activity by HF diets has been observed in several tissues.^28^, ^29^, ^49^, ^50^ On the other hand, it has been reported that GF mice fed a WD for 5 weeks have increased phospho‐AMPK, compared to their colonized counterparts. Thus, leading to the hypothesis that elevated AMPK activity in GF mice may contribute to enhanced insulin sensitivity and obesity resistance.^7^ Interestingly, in this study we could not detect a statistically significant difference in AMPK activation between GF and CONV mice. It is worth mentioning that in the current study, phospho‐AMPK levels were detected after a longer dietary exposure (16 weeks). Such protracted dietary challenge might also explain the observed trend towards reduced AMPK protein levels at the end of the dietary intervention, as it was previously shown that a chronic HF feeding likely affects AMPK at the gene expression level.^28^ Collectively, this evidence corroborates the idea that nutrient overload responses and consequent fat storage are functional also in the complete absence of the microbiota.

Building further on this idea, our results suggest that chronic WD feeding triggers adipose tissue inflammation also in the absence of a microbiota. In early 2000, a number of studies linked LPS and HF‐diet‐induced inflammation and metabolic dysfunction.^43^, ^51^, ^52^, ^53^, ^54^ Specifically, landmark findings in 2007 indicated modification in the gut microbiome as well as a moderate increase in the bacterial lipopolysaccharide (LPS) in the systemic circulation of mice fed a HF diet. Such low‐grade inflammation was termed “metabolic endotoxemia”.^43^ Thus, HF diet consumption increases gut barrier permeability allowing bacterial components into the blood stream and triggering the inflammatory state. Indeed, the same group reported that antibiotic treatment significantly decreases circulating LPS as well as gut permeability and adipose tissue inflammation, eventually resulting in improved metabolic markers of obesity and diabetes in mice fed a HF diet.^36^ The preliminary observations reported here are somewhat surprising and indicate that a calorie‐dense diet alone is sufficient to trigger an inflammatory response in adipose tissue.

Mechanistically, increased fatty acid oxidation was indicated as one of the explanations for DIO resistance in GF mice, whereby the activity of the mitochondrial protein CPT1 was elevated in gastrocnemius muscle and liver of these mice.^7^ Although our GF mice developed obesity, we also observed elevated CPT1 activity in gastrocnemius muscles of these animals, as compared to CONV controls. Although the literature indicates that alteration of mitochondrial fatty acid oxidation is an influencing factor of glucose homeostasis, the mechanistic link is unclear.^55^ For instance, seemingly contrasting findings indicate both the inhibition^56^, ^57^ and stimulation^58^ of muscle fatty acid oxidation to ameliorate insulin resistance.

Our present results suggest that dietary composition and mouse strain likely do not explain discrepancies among earlier studies. One could speculate that small genetic variations may occur within the same mouse strain and even more so, when mice are obtained from different laboratories. This could be a reason for this study being in contrast with the original study^7^ using the same mouse strain. It is appreciated that the host genotype is a contributing factor shaping the gut microbiota,^59^ accounting for potential differences in the response of our CONV mice to the same WD. However, a study by Hildebrandt *et al* indicates that changes in the gut microbiota seem to be driven by the diet and appear both in the presence and absence of the obese phenotype.^60^

Environmental factors might have played a role in the different outcomes of our studies. For instance, increased locomotor activity of GF mice was observed in the first study.^7^ As we did not investigate behaviour in the present study, we cannot exclude that our GF mice had a lower level of activity, thus, favouring the weight gain. However, increased locomotor activity is normally associated with increased lean mass and likely changes in food and water consumption, but we did not see any such differences between CONV and GF groups treated with either regular or WD.

One important difference between the present study and the original study is the use of conventionally raised mice rather than conventionalized mice as a control group. An adaptation period of about 2‐3 weeks has been recently described following colonization. During this phase, body weight, fat mass and glucose metabolism of the GF animal progressively shift towards a conventional‐like state.^61^ Nonetheless, conventionalized mice in the original study were switched to a WD 2‐3 weeks after colonization, thereby limiting the impact of this variable on the following 8‐week study.^7^

In conclusion, we demonstrate that the absence of a gut microbiota does not afford protection against diet‐induced obesity and metabolic dysfunction. Importantly, our findings do not contradict a crucial role of the microbiota in maintaining metabolic homeostasis as well as its importance in the onset and progression of metabolic diseases. Instead, we highlight the unequivocal, crucial role of dietary habits in preserving metabolic homeostasis.

## 4 MATERIAL AND METHODS

### 4.1 Animals

This study was approved by the Institutional Animal Care and Use Committee in Stockholm and performed according to the National Institutes of Health (NIH) guidelines and with the EU Directive 2010/63/EU for the conduct of experiments in animals.

Aged‐matched, male GF (n = 10) and CONV (n = 6) C57BL/6J mice were obtained from the breeding facility at Astrid Fagraeus Laboratory at the Karolinska Institutet (Stockholm, Sweden). When reaching 11 weeks of age, mice were switched to a Western Diet (40.7% of total calories from fat; 40.6% from carbohydrate; 18.7% from protein; TD96132, Envigo Teklad Diets, Madison; Table 1). Body weight, food and water intake were subsequently monitored every week for up to 16 weeks. For comparison, a group of age‐matched GF and CONV mice on a standard rodent diet is shown.

**Table 1:** Experimental diet composition

Formula	g/kg	
Casein	236.0	
DL‐Methionine	3.54	
Sucrose	182.62	
Corn Starch	160	
Maltodextrine	120	
Hydrogenated Vegetable Oil	100	
Beef Tallow	100	
Cellulose	40	
Mineral mix, AIN‐93G‐MX (90046)	41.3	
Calcium phosphate, dibasic	4.72	
Vitamin Mix, Teklad (40060)	11.8	
Ethoxyquin, antioxidant	0.02	
Selected Nutrient Information	% by weight	% kcal
Protein	20.9	18.7
Carbohydrate	45.5	40.6
Fat	20.2	40.7
Kcal/g 4.5		

All the GF mice in this study were given sterile food and water. Mice on a regular rodent diet received autoclaved food (R34; 4% of total calories from fat; 9.5% from carbohydrate of which 3.5% from crude fibber; 16.5% from protein, Lantmannen, Sweden). The Western diet used in this study was irradiated (37.4 kGy delivered dose) by the supplier. In fact, owing to their high fat content, Western diets would be destroyed if autoclaved. Therefore, this diet was purchased irradiated and contained in smaller (250g) double‐sealed packages.

The germ‐free status of the animals was assessed weekly according to standard procedures for a gnotobiotic facility.^62^ Briefly, faecal samples were collected from each cage containing 2 to 4 mice each and cultured both aerobically and anaerobically at +37°C. The plates were read for up to 2 weeks. All GF mice in this study were housed in aseptic isolators until the day of the terminal experiments, when all the in vivo procedures were performed within a few hours to prevent extensive bacterial contamination.

All animals were maintained on a 12 hours light/dark cycle and housed in a temperature and humidity‐controlled environment with free access to food and water.

### 4.2 In vivo procedures

At the end of the dietary intervention, GF and CONV mice were fasted for 6 hours before blood was collected from the tail vein and fasting blood glucose measured. The oral glucose tolerance test (OGTT) was performed by oral administration of 2 g/kg body weight of a 20% glucose solution (D‐Glucose, Sigma) followed by blood glucose measurements at 15, 30, 60 and 120 minutes. Subsequently, mice were anaesthetized with light isoflurane‐anaesthesia (Forene; Abbott Scandinavia AB, Solna, Sweden) and body composition was assessed by dual‐emission x‐ray absorptiometry (DEXA) by a Lunar PIXImus densitometer (GE Medical‐Lunar, Madison, WI, USA). Immediately afterwards, blood and organs of interest were collected, processed and frozen for later analyses. Organ weight was noted before any further processing of the tissue, whereas gastrocnemius muscles were dissected and kept in isolation media,^63^ on ice and at 4°C overnight before the mitochondria were isolated.

### 4.3 Hepatic triglycerides quantification

Frozen hepatic tissue (100 mg) was homogenized with a bullet blender device in 1 mL of 5% Nonidet P40 Substitute. In order to solubilize triglycerides, samples were slowly heated twice to 80‐100°C for 2‐5 minutes and then cooled to room temperature. Insoluble material was removed by centrifuging the homogenates at top speed for 2 minutes. Samples were finally diluted 10 folds in water and triglycerides quantified using a Triglyceride Colorimetric Assay kit (Cayman Chemical Company, #10010303) according to the manufacturer´s instructions.

### 4.4 Oil red O staining

Fresh liver tissue was embedded in OCT cryomount embedding medium (Histolab Products AB, Sweden) and immediately snap frozen. Frozen sections (10 µm) were fixated with 1% paraformaldehyde for 10 minutes at 4°C. After rinsing, sections were let air dry for a few minutes and absolute propylene glycol was added for 5 minutes. Sections were incubated with pre‐warmed Oil Red O solution (0.5% in absolute propylene glycol) for 10 minutes and differentiated in 85% propylene glycol for 3 minutes. After rinsing in distilled water, Mayer´s Hematoxylin (Sigma‐Aldrich) was added on each section for 30 seconds. Sections were then washed under running tap water for 3 minutes and mounted with glycerine mounting medium before images acquisition. Quantification of Oil Red O absorbance was performed with imageJ software.

### 4.5 Real‐time quantitative PCR

The mRNA from individual visceral adipose tissue of GF and CONV mice fed a regular diet (GF n = 4; CONV n = 6) or a Western diet (GF n = 6; CONV n = 6) for 16 weeks was extracted using Trizol reagent (ThermoFisher) according to the manufacturer’s instructions. cDNA was synthesized from 1 µg mRNA using a reverse transcription kit (Applied Biosystems Ref: 4368814). Expression of PAI‐1 gene was performed using SYBR Green (Applied Biosystems) real‐time quantitative PCR (7500 Fast Real‐Time PCR System, Applied Biosystems). Primers sequences for PAI‐1 are PAI‐1‐F: 5′‐CAGCCTTTGTCATCTCAGCC‐3′; PAI‐1‐R: 5′‐CCGAACCACAAAGAGAAAGGA‐3′. Final gene expression was calculated using the 2^−ΔΔCt^ method, relative to the level of RPL19 as housekeeping gene: RPL19‐F 5′‐GAAGGTCAAAGGGAATGTGTTCA‐3′; RPL19‐R: 5′‐CCTTGTCTGCCTTCAGCTTGT‐3′. Each sample was assessed as three technical replicates of 6‐4 biological samples per each condition.

### 4.6 Western blot analyses

Pieces of frozen liver tissue were weighed (about 10 mg) and transferred to ice‐cold RIPA buffer (100 µL) containing protease (1:1000 dilution; Thermo Scientific) and phosphatase inhibitors (1:100 dilution; Thermo Scientific). Zirconium oxide beads (0.5 mm) were added to each sample before homogenization at 4 degrees, with a bullet blender system. The homogenized were subsequently centrifuged at 10 000× *g* for 10 minutes at 4°C and the supernatant transferred to a new tube. Protein concentration was determined by Bradford assay and 40 µg of protein for each sample was separated on 4%‐20% SDS‐polyacrylamide gels by electrophoresis before transfer to polyvinylidene difluoride membranes. Membranes were incubated overnight at 4°C in 5% BSA/0.1% Tween‐20/PBS containing rabbit anti‐AMPKα (1:1000 final dilution; Cell Signaling Technology #2532); rabbit anti‐phospho‐AMPKα (1:1000 final dilution; Cell Signaling Technology #2535) or mouse anti‐vinculin (1:1000 final dilution; Abcam, ab129002). Blots were developed using SuperSignal West Femto Maximum Sensitivity Substrate (Thermo Scientific, Rockford, US) and intensities were quantified using densitometry (ChemiDoc MP Imaging System, BioRad).

### 4.7 Isolation of mitochondria

Gastrocnemius muscles were dissected, transferred to ice cold isolation media (sucrose 250 mM, HEPES 10 mM, EGTA 1 mM, BSA 1 g/L, pH 7,4) and kept on ice at 4°C overnight. On the following day, samples were weighted and roughly homogenized using a pair of scissors and further homogenized using a potter elvehjem homogenizer on a slush of ice in presence of proteinase (0.2 mg/mL). Homogenates were resuspended in 3‐ml isolation medium and centrifuged at 700× *g* for 10 minutes. The resulting supernatant containing the mitochondrial fraction was further centrifuged at 10 000× *g* for 10 minutes. Pellets were carefully washed in isolation medium in order to remove the buffy coat and recentrifuged at 7000× *g* for 5 minutes. After another washing step, pellets were diluted (0.3 μl mg^−1^ initial tissue weight) in preservation medium (EGTA, 0.5 mM; MgCl2, 3 mM; K‐lactobionate, 60 mM; taurine, 20 mM; KH2PO4, 10 mM; HEPES, 20 mM; sucrose, 110 mM; histidine, 20 mM; vitamin E succinate, 20 mM; glutathione, 3 µM; leupeptin, 1 µM; glutamate, 2 µM; malate, 2 µM; BSA 1 g/L and Mg‐ATP 2mM) and kept on ice until analysed.

### 4.8 CPT1 activity by high‐resolution respirometry

CPT1 activity was evaluated by high‐resolution respirometry (O2‐K, Oroboros, Austria). Mitochondrial respiration was measured in the presence of palmitoyl‐CoA (40 μM), I’carnitine (0.5 mM), malate (0.1 mM) and ADP (2.5mM). Respiration medium consisted of (mM) 0.5 EGTA, 3 MgCl_2_.6H_2_O, 60 K‐lactobionate, 20 taurine, 10 KH_2_PO_4_, 20 HEPES, 110 sucrose and 1 g/L BSA. DatLab7 (Oroboros) was used for data acquisition. Respiration was normalized to mitochondrial protein.

### 4.9 Statistical analyses

Data are presented as mean ± SEM unless otherwise stated. Statistical analyses were performed using *t* test or Mann‐Whitney test for comparisons between two groups and two‐way ANOVA with Sidak post‐test for multiple comparisons. A *P* value of less than .05 was considered statistically significant. All statistical analyses were performed with GraphPad Prism, version 8 (GraphPad Software).

## CONFLICT OF INTEREST

The authors have stated explicitly that there are no conflicts of interest in connection with this article.
